# Dietary Policies and Programs: Moving Beyond Efficacy and Into “Real-World” Settings

**DOI:** 10.1089/heq.2020.0050

**Published:** 2021-04-19

**Authors:** Stella S. Yi, Matthew Lee, Rienna Russo, Yan Li, Chau Trinh-Shevrin, Simona C. Kwon

**Affiliations:** ^1^ Department of Population Health, NYU School of Medicine, New York, New York, USA.; ^2^ Department of Sociomedical Sciences, Columbia Mailman School of Public Health, New York, New York, USA.; ^3^ Department of Population Health Science and Policy, Mt. Sinai Icahn School of Medicine, New York, New York, USA.

**Keywords:** nutrition, policy, program, public health, systems science, implementation science

## Abstract

**Purpose:** Dietary behaviors are key modifiable risk factors in averting cardiovascular disease (CVD), the leading cause of morbidity, mortality, and disability in the United States. Before investing in adoption and implementation, community-based organizations, public health practitioners, and policymakers—often working with limited resources—need to compare the population health impacts of different food policies and programs to determine priorities, build capacity, and maximize resources. Numerous reports, reviews, and policy briefs have synthesized across evidence-based policies and programs to make recommendations, but few have made a deep acknowledgment that dietary policies and programs are not implemented in a vacuum, and that “real-world” settings are complex, multifaceted and dynamic.

**Methods:** A narrative review was conducted of currently recommended evidence-based approaches to improving dietary behaviors, to describe and characterize applied and practical factors for consideration when adopting and implementing these dietary policies and programs across diverse settings.

**Results:** From the narrative review, six key considerations emerged to guide community-based organizations, public health practitioners, and policymakers on moving from the evidence base, toward implementation in local and community settings.

**Conclusions:** Considerations of “real-world” contextual factors are necessary and important when adopting and selecting evidence-based policies and programs to improve dietary behaviors and ultimately improve CVD outcomes. Promising approaches include those that apply community-partnered research and systems science to examine the equitable implementation of evidence-based dietary policies and programs.

## Introduction

Dietary behaviors are key modifiable risk factors in averting cardiovascular disease (CVD), a leading cause of morbidity and mortality in the United States.^1–3^ Despite global, national, and local initiatives to promote healthy dietary behaviors, building and sustaining healthy diets remain a difficult, perplexing health challenge. Most people do not meet, and are in fact far from meeting recommended dietary guidelines. Furthermore, evidence suggests modest or no changes in food consumption patterns in recent decades.^4,5^

Before investing in adoption and implementation, community-based organizations, public health practitioners, and policymakers—often working with limited resources—need to compare the health impact of different dietary policies and programs to determine priorities and maximize impact. Numerous works have synthesized across evidence-based policies and programs to make recommendations. What is lacking, however, is a deep acknowledgment that dietary policies and programs are not implemented in a vacuum, and that “real-world” settings are complex and ever-changing. The objective of this article is to present a brief summary of the currently recommended evidence-based policies and programs for improving diet and clearly delineate the applied and practical challenges in implementing dietary policies and programs that, while important, are not always presented. We conclude with recommendations for addressing these challenges and for advancing research on improving diets and dietary behaviors using approaches that integrate community-partnered research, system science, and implementation science.

## Guiding Frameworks for Dietary Policies and Programs: Historical, Reimagined, and Emerging

Multiple frameworks have been applied in public health to improve diet, including the social ecological model,^6,7^ the Geoffrey Rose model of population (vs. individual)-level intervention, and the Health Impact Pyramid as a framework for public health action.^8,9^ A cumulative result has been the support and adoption of a “Health in All Policies” approach at the multilateral, national, state, and local levels, which pushes for the consideration of health in policymaking across multiple sectors.

Pairing these frameworks with community partnerships has also become a global movement—with numerous examples from school-based nutrition programs, to community gardens and farmer's markets, to workplaces.^10^ It is worth highlighting a distinction, however, between the delivery of policies and programs in community settings compared to implementation utilizing principles of community-based participatory research (CBPR). CBPR is a collaborative approach to research that calls for the active and equal partnership of community stakeholders throughout the research process, starting first with a research topic that is of importance to the community.^11–13^ This point is raised not to undermine the importance of dietary policies and programs in communities that do not utilize formal CBPR methods, but rather to highlight a potential gap and key opportunity for future directions.

Two growing areas of research, systems science, and implementation science, are increasingly gaining traction in diet-related and obesity research.^14–21^ Systems science is a suite of computational research approaches that may be used to study and account for complex connections, feedback loops, and dynamic interactions between multilevel determinants of health and health outcomes. Population health researchers have used several types of systems science models—including system dynamics and agent-based modeling—to inform intervention design and policymaking.^22^ For diet specifically, systems science models have been utilized to study socioecologic mechanisms of the determinants of diet and dietary behaviors and policy-oriented impacts related to food pricing, advertising, and the food environment.^18^ Implementation science frameworks and methods—which seek to examine in context, how evidence-based interventions are adopted, implemented, sustained, and scaled across diverse settings^23^—are also relevant as they aim to optimize reach and impact, and are increasingly being recognized for their potential to contribute toward nutrition education.^24^

Several recent publications have suggested the theoretical promise and limitations of combining systems science and implementation science with CBPR and mixed-method data collection to advance health equity, understand the underlying causes of health disparities, and improve data science.^25–29^ Existing research utilizing this combined approach has mostly focused on tobacco and childhood obesity, indicating the opportunity for application of these methods to other health issues, including broader dietary programs and policies.

## The Current Evidence Base of Recommended Dietary Programs and Policies

Numerous peer-reviewed articles and reviews have summarized the breadth of population-level dietary programs and policies. The purpose of this article is not to provide a comprehensive review of the literature, but rather a contextualized overview of what dietary policies and programs are currently the most broadly supported evidence-based interventions and approaches. A narrative review was conducted between October 2018 and March 2019 of both peer-reviewed and gray literature on dietary policies and programs implemented globally over the past 10 years; additional detail may be found elsewhere.^10^ In scanning the vast literature covering these topics (*n*=489 relevant articles), four review sources were identified that, in addition to describing the specific approaches of existing dietary policies and programs, also provided a “score” as to whether the intervention had evidence to indicate both efficacy and effectiveness.

The evidence was synthesized across the four sources (Table 1) using the following process: (1) eight categories of dietary policy and program approaches were created based on the categories proposed in the sources and (2) whether the source recommended the approach (Table 2). The eight categories were as follows: multilevel interventions; food pricing strategies; nutrient-specific reformulation or elimination; mass media campaigns; reduce exposure and availability of unhealthy foods; community-based changes; direct consumer education; and food labeling. Overwhelmingly, three strategies stood out as being strongly supported by the research- and practice-based evidence: (1) multilevel strategies or approaches that included two or more strategies at multiple levels (e.g., individual-level nutrition education and school food policies); (2) food pricing strategies—such as taxation of unhealthy foods (e.g., sugary drink tax) or subsidies to lower prices of healthy foods (e.g., fruits and vegetables); and (3) nutrient-specific reformulation or elimination policies to reduce specific nutrients in foods (e.g., trans fats and sodium). Mass media campaigns, particularly those that targeted a single dietary factor or food, and reducing exposure and availability of unhealthy foods, had modest support from the evidence base. For example, Afshin et al. concluded that mass media campaigns may be effective in improving behaviors around fruit, vegetable, or salt consumption, but effectiveness of campaigns focused on other dietary targets has not been established.^30^ Moreover, in-depth investigations on factors such as intensity, duration, or potential effects on health disparities of campaigns have not been adequately conducted. The remaining three approaches (community-based changes, direct consumer education, and food labeling) were less strongly supported as having a meaningful impact on dietary outcomes and behaviors.

**Table 1.** Overview of the Four Sources

Lead organizations/authors	Years covered	Title	Summary points
The New York Academy of Medicine (NYAM) in partnership with the New York City Department of Health and Mental Hygiene (NYC DOHMH) Fisher and Griffin^58^	2000–2016	*Interventions for Health Eating and Active Urban Living: A Guide for Improving Community Health*	• Twenty-five described approaches • Eight Groupings: overarching approaches, multifaceted interventions, community-based nutrition interventions, make healthy foods more affordable, prioritize investment in local agriculture and procurement of local food products, promote healthy foods and beverages, increasing consumer education around eating, preparing and purchasing healthy foods, reduce exposure to unhealthy foods, beverages and eating practices • Three-category rating system for evidence: ○ Supportive evidence—policies and programs supported by at least one systematic review or at least two experimental studies or two quasi-experimental studies with matched concurrent comparisons ○ Emerging evidence—supported by no more than one experimental or quasi-experimental study with a matched concurrent comparison ○ Recommended by experts in the field of population health and chronic disease prevention
American Heart Association (2012)^2^	1980–2012	*AHA Scientific Statement Population Approaches to Improve Diet, Physical Activity, and Smoking Habits: A Scientific Statement from the American Heart Association*	• Three population health priorities (improve dietary habits, increase physical activity, and reduce tobacco use)—diet-related results only described in this review • Six groupings: media and education, labeling and information, schools, workplaces, local environment, restrictions and mandates • Classification of recommendations: ○ Three classes: • Class I—Intervention should be performed • Class IIa—It is reasonable to perform the intervention • Class IIb—The intervention may be considered • Class III—Intervention not useful/harmful ○ Weight of evidence: • Level of Evidence A—data derived from multiple randomized clinical trials, well-designed quasi-experimental studies • Level of Evidence B—data derived from a single randomized trial or nonrandomized studies • Level of Evidence C—only consensus opinion of experts, case studies, or standard of care
Afshin et al.^30^	1980–2015	*CVD Prevention Through Policy: a Review of Mass Media, Food/Menu Labeling, Taxation/Subsidies, Built Environment, School Procurement, Worksite Wellness, and Marketing Standards to Improve Diet*	• Six groupings: media and education, labeling and information, schools, workplaces, local environment, restrictions and mandates • Three-category rating system for evidence: ○ Supported ○ Mixed or inconclusive ○ Not enough evidence
Hyseni et al.^32^	1975–2015	*The Effects of Policy Actions to Improve Population Dietary Patterns and Prevent Diet-Related Non-communicable Diseases: Scoping Review*	• Scoping review (review of reviews) • Seven categories based on a social marketing framework: food price; food promotion; food provision; food composition; food labeling; food supply chain, trade and investment; multicomponent intervention • Four category-rating system for evidence ○ Consistently effective ○ Very effective ○ Less effective ○ Limited evidence

CVD, cardiovascular disease.

**Table 2.** Summary of Policies and Programs Supported by the Evidence Base

Strategy	Description	Sources			
		NYAM Report^58^	AHA^2^	Afshin et al.^30^	Hyseni et al.^32^
Multilevel interventions	Multilevel within schools, workplace; or, Inclusive of 2 or more strategies at multiple levels (e.g., individual-level nutrition education and school food policies)	+	+	+	+
Food pricing strategies	Taxation of unhealthy foods (e.g., sugary drink tax) Subsidies to lower prices of healthy foods	+	+	+	+
Nutrient-specific reformulation or elimination	Regulatory or voluntary policies to reduce specific nutrients in foods (e.g., trans fat and sodium)	N/A	+	N/A	+
Mass media campaigns	Targeting a single dietary factor or food	=	+	+	=
Reduce exposure and availability of unhealthy foods	Reduce advertisement of unhealthy foods Reduced availability and consumption of sugar-sweetened beverages and junk food	+	+	=	=
Community-based changes	School or community gardens Availability or promotion of healthy foods at small, local retailers; or in schools, including water Healthy vending machines Increased availability of supermarkets in communities with limited access to healthy foods	+	=	=	+
Direct consumer education	Taste testing fruits and vegetables Cooking programs Grocery-based educational programs	+	=	N/A	=
Food labeling	Nutrition panels Calorie labeling in stores/restaurants	N/A	=	=	=

Support denoted by + defined as strong supporting evidence found for one or all of the approaches listed. Findings that were less strongly supported or a cited lack of evidence by source denoted by=.

To ensure that results of overlapping studies were not being reported, citations from each of the four sources were examined. Less than 20% of the references overlapped. Of the 187 sources cited in Afshin et al., only 27 references were shared with the American Heart Association review and 4 references with the Hyseni et al. review. The Afshin et al. review did not share any source with the Hyseni et al. review, and the NYAM report did not share citations with any of the three other articles. Overall, these articles presented findings from a wide variety of articles and yet independently derived similar conclusions about effectiveness.

## Discussion: Key Considerations for Moving Beyond Evidence and Into Implementation

These four reviews provide an evidence-based foundation for policymakers and public health officials looking to adopt and implement dietary policies and programs. Yet, while there is some mention of factors related to practical decision-making and execution within these reviews, they focus primarily on policies, with community-based programs receiving less attention. Below, key considerations salient to “real-world” implementation of dietary policies and programs, which should be weighed together with evidence-based recommendations, are summarized.

1. What contextual factors will affect implementation? There are inner and outer contextual factors that potentially affect dietary policy and program implementation, including intervention cost and feasibility in specific settings. For example, a community-based organization with a modest budget will have limited capacity to solely implement a new multilevel nutrition-related intervention or program that targets food pricing strategies compared to consumer education activities—which rates lower on the effectiveness scale according to the evidence base. This points toward opportunities to partner with multisector institutions like academia or government to achieve community-wide implementation and sustainability. Organizational support (e.g., availability of staff and staff workload), social relationships and support (e.g., collaboration across groups, networks, visions, conformity, and identity in groups), and leadership (e.g., influences on managers, change agents, opinion leaders, and program champions) have also been identified as common implementation factors.^23^ Implementation-related factors are cited as important within the realm of dietary policy and programs at all levels from community to goverment,^31^ in particular because a failure to consider context may lead to the premature dismissal of a policy or program as being ineffective or unsustainable. Despite this, there are little to no examples of application of implementation science frameworks within the dietary policy and program literature.
2. What are the unintended consequences of dietary policies and programs? A handful of articles identified the potential for unintended consequences of dietary policies,^14,32,33^ such as the replacement of trans fatty acids in food reformulation strategies with fats and oils high in saturated fats^34^; or for sugary drink taxes to increase consumption of alcohol.^35^ More broadly, because large-scale public health programs and policies can have unintended consequences, it becomes important to understand which implementation factors influence the intervention components that are either working or not working as intended, as well as for whom and under what circumstances.^36^ Recently, a definition was advanced for “equitable implementation” that still needs to be operationalized, and dietary policies and programs are a promising area to examine whether integrating strong equity components into implementation science tools can facilitate higher quality, and more even implementation of effective policies and programs across communities, especially among those that experience persistent CVD disparities. Without attention to equitable implementation, policies may perpetuate stigma toward subgroups of the population as in the case with obesity policy—where parents of obese children are directly or indirectly blamed^33^ or individuals themselves are at higher risk for weight-based bullying or disordered eating.^37^ Least optimally, some dietary policies and programs and their implementation may actually widen disparities across socioeconomic or racial/ethnic minority groups.^4,38^ In a recent review, dietary programs focused on “price” strategies were identified to have the most potential for decreasing dietary disparities, while “person” strategies, especially dietary counseling, were likely to increase disparities.^38^ There is little attention with regard to unintended consequences of dietary programs, and more importantly, work toward a systematic method for assessing, managing, acting upon, and adjusting for such consequences in subsequent initiatives.
3. What is the public's opinion about different approaches? Public opinion on dietary programs and policies has been well evaluated in peer-reviewed and gray literature—and has been argued in some cases, to not be relevant for policy decision-making.^37^ For example, support for sugary drink taxes in the U.S. is moderately low at 35%^39^ (although this does vary by region), yet seven cities and the Navajo Nation have implemented such taxes.^40^ In some cases, the public may support a strategy, but the effectiveness of the policy in terms of changing behavior is low. For instance, introducing new supermarkets (77%) into low-resourced neighborhoods is highly supported,^41^ yet their new presence does not appear to impact diet quality.^42^ Under other circumstances, public support for an approach may exist, funder support may exist, but the utility for the target community may be limited. For example, while nearly half of U.S. adults support community gardens, and hundreds have been funded and implemented over the past several years, a lack of community interest was cited as a key barrier to the success of community gardens in a recent systematic review.^43^ Public opinion can be an important factor in implementation successes and failures because it not only assesses community-level accord or discord, which can be used to identify the potential to leverage collective efficacy for action, but also can be a factor to influence decision-maker attitudes and behaviors (e.g., continued attention/support for a specific policy approach or public health issue). Some policy dissemination efforts have focused on evidence-based mental health programs, and dietary policies and programs are also a promising area for similar research.^44^
4. What is a reasonable timeframe for policy and program adoption, implementation, or evaluation? A major challenge of dietary policy and program implementation and evaluation is the timeline. Adoption and implementation of taxes or other systems-level strategies may take years, and must align with a favorable window of opportunity in the political or policy environment. Furthermore, the public health benefits of policies and programs are often dispersed and delayed, making it difficult to attribute causality or linkage between a particular policy/program and dietary behavior change, especially because policy processes move more quickly than research and evaluation processes do (e.g., by the time findings support the continued implementation and sustainment of a program or policy, the political and social climate have changed).^45^ Issues arise with regard to evaluation as well, including informal implementation of policies before an official “start date” or on a microlevel, the seasonality of dietary behaviors and availability of fresh produce over the course of a calendar year.^46^
5. What are alternative outcomes to consider, besides health? It has been suggested that due to the long lag time of chronic disease, more proximal measures, such as awareness of programming, social norms, or behaviors of both the individual and the target population are important and complementary factors to clinical outcomes in epidemiologic studies.^2^ Social norms in particular may be a critical cornerstone to understanding the underlying causal framework of the effects that dietary policies and programs have on behaviors, and ultimately disease end-points. Some of the observed effects of public health campaigns have been attributed to shifting social norms—although not the intended target of the campaigns. For example, the dramatic declines in tobacco use may be attributed to the cultural shift in norms from “accommodation to intolerance” of public smoking.^47^ More recent conceptualization of the influence of social norms has been expanded to “social exposure,” defined as the “composite of ways in which people come in contact with or experience a particular product or behavior in their environment”—which may include that person's social, physical, and symbolic environments.^48^ Despite the seeming importance of consideration of social norms, characterization of how these may change or have changed in relationship to dietary policies and programs does not appear to exist in this literature. Innovative methods in systems science are especially well suited to account for these important factors that contribute to both intervention and implementation effectiveness and success of dietary programs and policies. For example, agent-based modeling can account for the spread of social norms, and social network analysis can examine how beliefs and behaviors spread through networks across community settings and jurisdictions.^49^
6. Is the dietary program or policy sustainable? Dietary programs require a continued behavior change and that requires multilevel approaches to reinforce the behavior change not only at the individual level but also at the community and neighborhood levels, as well as continued resource allocation and prioritization at the policy and environmental levels. As such, greater attention needs to be paid to monitoring the continued implementation and enforcement of dietary policies, as well as the continued fidelity to program protocols, while also rigorously documenting both planned and unplanned adaptations over time.^50^ In implementation science, growing attention toward program and policy sustainability, as well as to the termination and de-implementation of interventions, highlights the need to allow promising interventions to achieve full initial implementation for a sufficient period of time (e.g., at least 1 year) before evaluating implementation effectiveness and sustainability.^51^ Furthermore, there is the growing recognition that sustainability is not a static end goal, but involves dynamic processes and outcomes that likely require adaptation over time to evolve with changing contexts, settings, and populations. The Afshin et al. review briefly discusses the importance of equitable and sustainable impacts—highlighting an Australian mass media TV campaign that monitored effects in the first year of implementation and what was sustained after, as well as a Norwegian free school fruit program that was evaluated for sustained impacts both in the first year and the third year following full initial implementation. More research is needed to not only understand the equitable sustainability of dietary programs and policies in additional settings, particularly less-resourced ones, but also to highlight the importance of cultural adaptation, for community and neighborhood settings.

## Implications for Research and Practice

A promising way to address these complexities related to dietary policies and programs would be an approach that combines aspects of CBPR, systems science, and implementation science.

For example, CBPR and implementation science approaches would address feasibility and sustainability issues and to some degree, public opinion by deeply embedding the research process within the community space and increasing community capacity. Systems science methods would allow for consideration of unintended consequences, the spread of social norms, and importantly, allow the simulation of multiple programs and policies at one time and at different intervention intensities (e.g., more/less community engagement, higher vs. lower taxes, and frequency of mass media efforts). Furthermore, systems science integrated with community-based mixed methods data collection would allow simulated effects of dietary programs and policies contextualized within the lived experience of community members, potentially accelerating the translational research trajectory. Importantly, such an approach may help to mitigate potential widening of health disparities across population subgroups.

To the authors' knowledge, only two examples of the application of CBPR, systems science, and implementation science exist in the current literature—both pertaining to childhood obesity in cities in the United States. The first example applied a combined approach toward the design and implementation of interventions to enhance healthy offerings at carry-out shops and corner stores in Baltimore, MD.^52^ The authors utilized previously collected and published intervention data and newly collected qualitative data to create an underlying causal loop diagram and then developed a systems dynamics model to understand factors affecting adoption, implementation, and maintenance (AIM of the Reach, Effectiveness, Adoption, Implementation, Maintenance [RE-AIM] framework)^53^ of the interventions.^52^ The authors identified stakeholder (storeowner) motivation and early and continued communication between the interventionist and stakeholder to be the most critical elements for intervention success, and provide specific details on how these may be operationalized in the practice setting.^52^ Moreover, the projects described represent a deep, trusted, and longstanding partnership in research between the academic institution and the community^54^—thus more meaningfully reflecting the priorities of the target population instead of merely being in the community setting.

In the second example, the research team described Shape Up Under 5, a 2-year early childhood obesity prevention pilot study,^55,56^ again borne out of a rich and longstanding partnership between stakeholders in the town of Somerville, MA, and academic researchers (Shape Up Somerville 2003-05).^57^ The team developed the Stakeholder-driven Community Diffusion conceptual framework, which combined community-engaged research with concepts of systems science (i.e., agent-based modeling, group model building, and social networks analysis) woven throughout with a goal of identifying and implementing a community-driven approach toward reducing childhood obesity.^55^ Both of these examples not only illustrate successful integration across CBPR, systems science, and implementation science but also reflect CBPR performed well, that is in promoting increased trust between researchers and communities, enhanced quantity and quality of collected data, and meaningful sustainability and impact.

To close, an integrated approach offers us a way forward to work collectively across sectors and across health disciplines in a way that reflects the needs of the community to improve diet and health and advance health equity.

## Abbreviations Used

CBPR: community-based participatory research
CVD: cardiovascular disease
